# Characterization of the metabolic profile associated with serum 25-hydroxyvitamin D: a cross-sectional analysis in population-based data

**DOI:** 10.1093/ije/dyw222

**Published:** 2016-09-07

**Authors:** Susanne Vogt, Simone Wahl, Johannes Kettunen, Susanne Breitner, Gabi Kastenmüller, Christian Gieger, Karsten Suhre, Melanie Waldenberger, Jürgen Kratzsch, Markus Perola, Veikko Salomaa, Stefan Blankenberg, Tanja Zeller, Pasi Soininen, Antti J Kangas, Annette Peters, Harald Grallert, Mika Ala-Korpela, Barbara Thorand

**Affiliations:** ^1^Institute of Epidemiology II, Helmholtz Zentrum München, German Research Center for Environmental Health, Neuherberg, Germany,; ^2^German Center for Cardiovascular Research (DZHK e.V.), Munich Heart Alliance, Munich, Germany,; ^3^Research Unit of Molecular Epidemiology, Helmholtz Zentrum München, German Research Center for Environmental Health, Neuherberg, Germany,; ^4^German Center for Diabetes Research (DZD e.V.), München-Neuherberg, Germany,; ^5^Computational Medicine, University of Oulu and Biocenter Oulu, Oulu, Finland,; ^6^NMR Metabolomics Laboratory, University of Eastern Finland, Kuopio, Finland,; ^7^National Institute for Health and Welfare, Helsinki, Finland,; ^8^Institute of Bioinformatics and Systems Biology, Helmholtz Zentrum München, German Research Center for Environmental Health, Neuherberg, Germany,; ^9^Department of Physiology and Biophysics, Weill Cornell Medical College in Qatar, Education City, Doha, Qatar,; ^10^Institute of Laboratory Medicine, Clinical Chemistry and Molecular Diagnostics, University Leipzig, Leipzig, Germany,; ^11^Institute for Molecular Medicine (FIMM) and Diabetes and Obesity Research Program, University of Helsinki, Helsinki, Finland,; ^12^Estonian Genome Center, University of Tartu, Tartu, Estonia,; ^13^University Heart Center Hamburg, Clinic of General and Interventional Cardiology, Hamburg, Germany,; ^14^German Center for Cardiovascular Research (DZHK e.V.), Partner Site Hamburg/Lübeck/Kiel, Hamburg, Germany and; ^15^Computational Medicine, School of Social and Community Medicine, University of Bristol and Medical Research Council Integrative Epidemiology Unit at the University of Bristol, Bristol, UK

**Keywords:** 25(OH)D, vitamin D, metabolomics, obesity, molecular epidemiology

## Abstract

**Background:** Numerous observational studies have observed associations between vitamin D deficiency and cardiometabolic diseases, but these findings might be confounded by obesity. A characterization of the metabolic profile associated with serum 25-hydroxyvitamin D [25(OH)D] levels, in general and stratified by abdominal obesity, may help to untangle the relationship between vitamin D, obesity and cardiometabolic health.

**Methods:** Serum metabolomics measurements were obtained from a nuclear magnetic resonance spectroscopy (NMR)- and a mass spectrometry (MS)-based platform. The discovery was conducted in 1726 participants of the population-based KORA-F4 study, in which the associations of the concentrations of 415 metabolites with 25(OH)D levels were assessed in linear models. The results were replicated in 6759 participants (NMR) and 609 (MS) participants, respectively, of the population-based FINRISK 1997 study.

**Results:** Mean [standard deviation (SD)] 25(OH)D levels were 15.2 (7.5) ng/ml in KORA F4 and 13.8 (5.9) ng/ml in FINRISK 1997; 37 metabolites were associated with 25(OH)D in KORA F4 at *P* < 0.05/415. Of these, 30 associations were replicated in FINRISK 1997 at *P* < 0.05/37. Among these were constituents of (very) large very-low-density lipoprotein and small low-density lipoprotein subclasses and related measures like serum triglycerides as well as fatty acids and measures reflecting the degree of fatty acid saturation. The observed associations were independent of waist circumference and generally similar in abdominally obese and non-obese participants.

**Conclusions:** Independently of abdominal obesity, higher 25(OH)D levels were associated with a metabolite profile characterized by lower concentrations of atherogenic lipids and a higher degree of fatty acid polyunsaturation. These results indicate that the relationship between vitamin D deficiency and cardiometabolic diseases is unlikely to merely reflect obesity-related pathomechanisms.

**Key Messages**

- We examined the serum metabolite profile associated with serum 25-hydroxyvitamin D levels in metabolites obtained from both a nuclear magnetic resonance spectroscopy (NMR)- and a mass spectrometry (MS)-based platform.
- The discovery, based on 415 metabolites, was conducted in 1726 participants of the population-based KORA-F4 study. The results were replicated in 6759 participants (NMR) and 609 (MS) participants, respectively, of the population-based FINRISK 1997 study.
- In all, 37 metabolites were associated with 25(OH)D in KORA F4 after correction for multiple testing. Of these, 30 associations were replicated in FINRISK 1997.
- Higher serum 25-hydroxyvitamin D levels were associated with a serum metabolite profile characterized by lower concentrations of atherogenic lipids and a higher degree of fatty acid polyunsaturation. These associations were independent of waist circumference and similarly observed in abdominally obese and non-obese participants.
- Our results indicate that the relationship between vitamin D deficiency and cardiometabolic diseases is unlikely to merely reflect pathomechanisms related to obesity.

## Background

Vitamin D has traditionally been recognized for its important role in bone biology,^1^ but research within the past two decades has started to focus on potential risks of vitamin D deficiency beyond skeletal health.^2^ In order to carry out its biological functions, vitamin D, which is either synthesized in the skin upon exposure to ultraviolet B radiation or taken up from the diet, is initially converted to 25-hydroxyvitamin D [25(OH)D] in the liver.^3^ Serum levels of 25(OH)D, the major circulating vitamin D metabolite,^3^ are the preferred measure to determine vitamin D status.^4^ In a strictly regulated process, 25(OH)D is hydroxylated to 1,25-dihydroxyvitamin D [1,25(OH)_2_D], the biologically active vitamin D metabolite, in the kidneys.^1^ With the exception of patients with kidney disease,^5^ oral vitamin D administration increases the circulating concentrations of both 1,25(OH)_2_D and 25(OH)D.^6^

Low levels of serum 25(OH)D are common throughout the European population.^7^ In Germany, 31% of the adult participants of the national health survey 2008-11 had inadequate levels of serum 25(OH)D (< 20 ng/l), and another 30% were 25(OH)D-deficient (< 12 ng/ml).^8^ Possible adverse effects of vitamin D deficiency on non-skeletal health are thus of considerable public health interest. Meta-analyses of observational studies have shown an association between low levels of serum 25(OH)D and a wide array of chronic diseases, including cardiovascular disease and type 2 diabetes.^2^^,^^9^ By contrast, findings from randomized controlled trials so far do not support a beneficial effect of vitamin D supplementation on cardiometabolic health.^2^^,^^9^ Previous trials were generally limited by factors such as inadequate study designs, low doses of vitamin D and relatively short follow-up periods.^10^ However, the discrepancy between observational and intervention studies on the relationship between vitamin D and cardiometabolic diseases may also result from the former being affected by residual confounding; 25(OH)D levels are known to be especially low in obese individuals,^11^ with the proportion of subjects with inadequate 25(OH)D levels being about twice as high in obese than in non-obese persons.^12^ Since obesity is also associated with type 2 diabetes and various cardiovascular diseases,^13^ a confounding effect by obesity seems relatively likely. However, associations between 25(OH)D and cardiometabolic risk factors have also been found in spite of adjustment for body mass index (BMI).^14–16^

A better understanding of the biological mechanisms related to vitamin D and a characterization of possible differences in the metabolic pathways associated with 25(OH)D levels in obese and non-obese individuals may help to untangle the relationship between vitamin D, obesity and cardiometabolic health. Metabolomics, the comprehensive analysis of measurable metabolites in biological samples^17^ (ranging from low-molecular-weight metabolites to large metabolite complexes^18^), has provided new insights into the development of chronic diseases.^17^ Coupled with traditional epidemiology, metabolomics offers the potential to study diseases in humans more comprehensively, and it has already been successfully applied in the research on diseases such as atherosclerosis, diabetes and obesity.^19^ So far, however, only few studies have used metabolomics to examine the link between vitamin D and health or the metabolic profile associated with vitamin D, in a limited number of participants from very selected populations.^20^^,^^21^

In the present study, we used population-based data to characterize the serum metabolite profile associated with serum 25(OH)D levels, applying metabolomics obtained from two platforms based on mass spectrometry (MS) and nuclear magnetic resonance (NMR) spectroscopy. Furthermore, we aimed to assess possible differences in the metabolic signature of serum 25(OH)D levels in abdominally obese and non-obese participants.

## Methods

### Study populations

KORA (Cooperative Health Research in the Region of Augsburg) is a research platform for population-based surveys and subsequent follow-up studies in community-dwelling individuals living in the region of Augsburg in Southern Germany.^22^ The present study is based on data from the KORA F4 study, which was conducted between 2006 and 2008 as a follow-up of the KORA survey S4. A total of 3080 of the 4261 KORA S4 participants, aged 32–81 years at the time of the follow-up, took part in KORA F4. More details on the sampling method are given elsewhere.^22^^,^^23^ The replication was done in FINRISK 1997, a general population study conducted to monitor the health of the Finnish population among persons aged 24–74 years at recruitment.^24^ In total, 8444 individuals were recruited to represent the working-age population of five study areas across Finland.^24^ Both the KORA F4 study and the FINRISK 1997 study were carried out in accordance with the Declaration of Helsinki and approved by the ethics committees of the Bavarian Medical Association and the National Public Health Institute of Finland, respectively. Written informed consent was obtained from all participants.

### Data collection

In KORA F4, all participants underwent a standardized medical examination and a computer-aided personal interview at the study centre, which were conducted by trained examiners and interviewers. Information on socio-demographic variables, physical and outdoor activity, smoking behaviour, alcohol consumption, comorbidities, self-rated physical constitution and medication use was obtained in the interview. Leisure time physical activity in summer and winter^25^ was dichotomized in ‘inactive’ (less than 1 h of regular activity per week in summer and winter) and ‘active’.^26^ Additionally, the amount of time participants usually spent outdoors in summer was recorded.^27^ Alcohol consumption was calculated from the intake of beer, wine and spirits during the previous week.^28^ Comorbidities included diabetes mellitus (defined by self-report or current use of antidiabetic drugs) as well as having a history of myocardial infarction, stroke or cancer (inpatient treatment, self-reported). Physical constitution was assessed with the question: ‘How would you rate your current physical constitution?’. Furthermore, participants were asked to bring the product packaging of all medications they were currently using to the study centre. Based on this, intake of medication during the previous week was recorded through database-supported computer software.^29^ Height, weight and waist circumference were measured in light clothing. Height was measured to the nearest 0.1 cm, weight to the nearest 0.1 kg. Waist circumference (WC) was measured at the mid point between the lower rib and the upper margin of the iliac crest to the nearest 0.1 cm. Body mass index (BMI) was calculated as weight in kilograms divided by height in metres squared. In FINRSIK 1997, standard clinical laboratory measures were collected, and participants filled out questionnaires on physical activity. Physical activity was defined by the combination of two questions: ‘How much time do you usually spend on spare time physical activity?’ (‘active’: 1 h or more) and ‘At how many times per week do you usually do spare time physical exercise?’ (‘active’: once per week or more). Volume of ethanol consumed in the past week was calculated from beverage-specific questions on consumption of beer, cider and long drinks, wine and spirits.

### Laboratory analyses

In KORA F4, blood samples were collected between 8 a.m. and 11 a.m. after an overnight fast of at least 8 h. For serum collection, blood was drawn into serum gel S-Monovette tubes (Sarstedt, Nümbrecht, Germany), gently inverted two to three times and rested for 30 min at room temperature to obtain complete coagulation. Subsequently, the tubes were centrifuged for 10 min (2750 *g* at 15°C) and the serum was aliquoted into synthetic straws which were kept for a maximum of 6 h at 4°C before storage at −80°C until analysis. Total 25(OH)D concentration in the serum was measured using a chemiluminescent immunoassay (LIAISON 25 OH Vitamin D TOTAL Assay, DiaSorin Inc., Stillwater, USA). The inter-assay coefficients of variation were 8.7% and 9.1% for target values of 14.7 ng/ml and 47.5 ng/ml, respectively. One participant with an outlying 25(OH)D concentration (values outside mean ± 5 standard deviations) was excluded from the data set. The season of blood sampling was categorized according to the meteorological season. This classification of season has been used in the KORA F4 study before.^27^ Serum creatinine concentration was assessed with a modified Jaffe test (Krea Flex; Dade Behring), and the estimated glomerular filtration rate was calculated using the CKD-EPI equation.^30^ The liver enzyme gamma-glutamyl-transferase was measured on a Roche/Hitachi cobas c system, in accordance with the recommendations of the International Federation of Clinical Chemistry. In FINRSIK 1997, 25(OH)D_3_ was measured with the ARCHITECT system (Abbott Laboratories) that uses a chemiluminescent microparticle immunoassay, which is highly sensitive to natural 25(OH)D_3_ but less so to synthetic 25(OH)D_2_.^31^

### Discovery step in KORA F4; Metabolomics analyses

As described in our previous work,^32^ metabolite identification and quantification in the serum were performed on two different platforms, based on MS (Metabolon Inc., Durham, NC, USA)^33^^,^^34^ and NMR spectroscopy,^18^^,^^35^ respectively. The commercial MS platform is based on two ultra-high-performance liquid chromatography/tandem mass spectrometry (UHPLC/MS/MS2) injections and one gas chromatography/mass spectrometry (GC/MS) injection per sample. The two UHPLC injections were optimized for basic and acidic species, respectively (see^33^ and^34^ for more details). The platform provided relative quantification for a total of 517 compounds, 344 of which could be identified based on an in-house library of retention index, mass to charge ratio and fragmentation spectra. The data were pre-processed and corrected for batch effects as described before.^32^ For each metabolite, outlying values (values outside mean ± 4 standard deviations on the log_10_ scale) were removed from the data set; 81 metabolites with more than 50% missing values as well as 2 duplicate metabolites were excluded, leaving 434 metabolites (296 identified and 138 unidentified).

Additionally, an NMR spectroscopy platform^18^^,^^35^^,^^36^ was used to obtain quantitative information on 130 metabolites and derived measures. Outlier exclusion and detection rate calculations were performed as described for the MS data; 11 redundant measures representing sums or differences of other metabolites were excluded, leaving 119 metabolites; And 20 metabolites were covered by both the MS and the NMR spectroscopy platforms. These metabolites were treated as independent, that is the respective metabolites from both platforms were included in the analyses. More detailed considerations on the combined analysis of metabolites measured on the two metabolomics platforms are described elsewhere.^32^

In total, 415 metabolites and derived measures (296 identified metabolites measured with MS as well as 119 metabolites measured with NMR spectroscopy), were used for the association analyses. These metabolites cover several metabolite classes, including constituents of lipoprotein subclasses, fatty acids, glycerophospholipids, steroids, acylcarnitines, bile acid metabolites, amino acids, small peptides, carbohydrates, xenobiotics, vitamins and nucleotide metabolites. A full list of all metabolites can be found in the Supplementary Table S1, available as Supplementary data at *IJE* online. Throughout the paper, (M) and (N) are used to indicate metabolites derived from the MS and the NMR spectroscopy platform, respectively.

### Discovery step in KORA F4; Statistical analyses

Data from both metabolomics platforms were available in a subsample of 1745 participants. In addition to one participant with an outlying 25(OH)D concentration, we excluded: nine participants with more than 20% missing values in at least one of the metabolomics platforms; eight participants who were pregnant or had a fasting duration of less than 8 h at the time of the examination; and one participant with missing information in the covariables; leaving a study population of 1726 participants. For missing data treatment, we chose multiple imputation by chained equations.^37^ The imputation strategy is described in the Supplementary material, available as Supplementary data at *IJE* online. The distribution of the metabolites was tested against normality before imputation, followed by transformation where necessary (Supplementary Table S2, available as Supplementary data at *IJE* online). Metabolites were standardized to a mean of zero and a standard deviation of one. Multiple linear models were used to assess the association between each metabolite (outcome) and continuous 25(OH)D levels (predictor). In the main analysis, the models were adjusted for age, sex, season, WC (continuous), physical activity, time spent outdoors, smoking status and alcohol consumption. The Bonferroni method was used to account for multiple testing. Thus, the adjusted *P*-value threshold was set at 1.20 x 10^−4^ (0.05/415). To assess possible differences in the associations of 25(OH)D with the metabolites between abdominally obese and non-obese subjects, the main models were stratified by WC status. For this, the cut-off for abdominal obesity was set at a WC ≥ 88 cm in women and ≥ 102 cm in men. Formally, the main models were extended by interaction terms of the WC status with 25(OH)D and with each of the other covariables. In these models, the main effect estimate of 25(OH)D and the sum of the main effect estimate of 25(OH)D plus the 25(OH)D:WC interaction effect can be interpreted as the association of 25(OH)D with the metabolite concentration in the WC reference category and in the alternative category, respectively. Both effects were tested using linear hypothesis tests. The adjusted *P*-value threshold was set at 6.02 x 10^−05^ (0.05/(2*415)). All regression analyses were performed with SAS 9.3 (SAS Institute Inc., Cary, NC, USA).

### Replication step in FINRSIK 1997

Metabolite identification and quantification in the serum was conducted in accordance with KORA F4, in 7503 individuals (NMR) and 682 individuals (MS), respectively. The MS data were batch-corrected and scaled for the analyses. The metabolites were transformed according to the normal transformations used in KORA F4. All analyses were performed on the complete observations for the respective metabolite. Data for both NMR and 25(OH)D were available for a maximum of 6759 participants, and data for both MS and 25(OH)D for a maximum of 609 participants. The 37 associations with 25(OH)D at *P* < 1.20 x 10^−4^ (Bonferroni threshold), which were identified in KORA F4, were replicated in FINRISK 1997. Thus, the *P*-value threshold for the replication was set at 1.35 x 10^−3^ (0.05/37). The models were adjusted for the same covariables as in KORA F4, except for time spent outdoors which was not available in FINRISK 1997, and season as the FINRISK 1997 samples were all collected during spring. Furthermore, as the median fasting time was only 5 h, time from the latest meal in hours was used as a covariable. For the stratified analyses, WC was categorized as in KORA F4, and the main model was repeated in both WC strata. All analyses in FINRISK 1997 were conducted with RStudio version 0.99.473 that uses R version 3.2.3.

### Sensitivity analyses in KORA F4

A number of the metabolites were highly correlated. Joint testing of clusters instead of single variables helps to avoid redundancy of the association results and increased power and reliability in earlier omics studies.^38^^,^^39^ Thus, in addition to the individual metabolite analyses, we examined eight modules of closely connected metabolites which had previously been identified by weighted correlation network analysis.^32^ The module membership of each metabolite is given in Supplementary Table S4, available as Supplementary data at *IJE* online. Associations between 25(OH)D and the metabolite modules could not be confirmed in the replication cohort. As the Metabolon MS platform is untargeted, the metabolites that were detected in KORA F4 and in FINRISK 1997 differed to some extent. Thus, the metabolite modules could not be calculated in FINRISK 1997. More details on the metabolite module analyses are given in the Supplementary material, available as Supplementary data at *IJE* online.

The main models for those metabolites that were associated with 25(OH)D at *P* < 1 .20 x 10^−4^ were re-examined using vitamin D quartiles. Tests for linear trends across quartiles were conducted by assigning the median value within each quartile to the respective quartile and by treating the assigned median values as a continuous variable. To assess the impact of WC as an operationalization of obesity, the main models were re-examined using BMI instead of WC. To assess whether the association between 25(OH)D and the serum metabolites is mediated by parathyroid hormone, the main models were additionally adjusted for parathyroid hormone. The influence of additional adjustment for diseases and physical constitution or intake of medication was examined in additional sensitivity analyses. In the disease model, the main models were additionally adjusted for diabetes mellitus, history of myocardial infarction, stroke or cancer, self-rated physical constitution, estimated glomerular filtration rate (as a measure of kidney function) and gamma-glutamyl-transferase (as a measure of liver function). In the medication model, the disease models were additionally adjusted for intake of oral contraceptives, estrogens, diuretics, statins, antiepileptic and oral antidiabetic medication, corticoids and calcium channel blockers as well as for angiotensin-converting enzyme inhibitors or angiotensin antagonists.

## Results

The characteristics of KORA F4 and FINRISK 1997 are shown in Table 1

**Table 1.** Characteristics of the study populations

		KORA F4	FR97 NMR	FR97 MS
Characteristic		*N* = 1726	*N* = 6762	*N* = 609
25(OH)D	ng/ml	15.2 (7.5)*	13.8 (5.9)*	14.1 (6.0)*
Age	years	60.8 (8.8)*	47.8 (13.0)*	50.7 (13.7)*
Sex	male	834 (48.3)	3352 (49.6)	381 (62.6)
	female	892 (51.7)	3410 (50.4)	228 (37.4)
Waist circumference	cm	95.6 (14.1)*	87.9 (13.4)*	90.6 (13.2)*
Waist circumference status^a^	abdominally obese	857 (49.7)	1665 (24.6)	172 (28.2)
	abdominally non-obese	869 (50.3)	5097 (75.4)	437 (71.8)
Time spent outdoors in summer	h/day	4.4 (2.5)*	–	–
Season	December–February	550 (31.9)	–	–
	March–May	408 (23.6)	–	–
	June–August	269 (15.6)	–	–
	September–November	499 (28.9)	–	–
Physical activity	active	995 (57.7)	1903 (28.1)	161 (26.4)
	inactive	731 (42.3)	4859 (71.9)	448 (73.6)
Alcohol consumption	g/day	15.5 (20.4)*	9.3 (16.9)*	9.9 (16.8)*
Smoking status	never	730 (42.3)	3591 (53.1)	305 (50.0)
	former	743 (43.1)	1548 (22.9)	141 (23.2)
	current	253 (14.6)	1623 (24.0)	163 (26.8)

Numbers indicate *N* (%) unless marked otherwise; *numbers indicate mean (standard deviation).

WC, waist circumference; 25(OH)D, 25-hydroxyvitamin D.

^a^Abdominally obese: WC ≥ 88 cm in women / ≥ 102 cm in men; abdominally non-obese: WC < 88 cm in women / < 102 cm in men.

; 25(OH)D levels were relatively low in both cohorts, with mean values being 15.2 ng/ml (2.0–46.9 ng/ml) in KORA F4 and 13.8 ng/ml (0.7–32.6 ng/ml) in FINRISK1997. The distributions of the 25(OH)D levels in both cohorts are shown in the Supplementary Figure S2a and b, available as Supplementary data at *IJE* online.

### Individual metabolites associated with 25(OH)D

**Figure 1.**
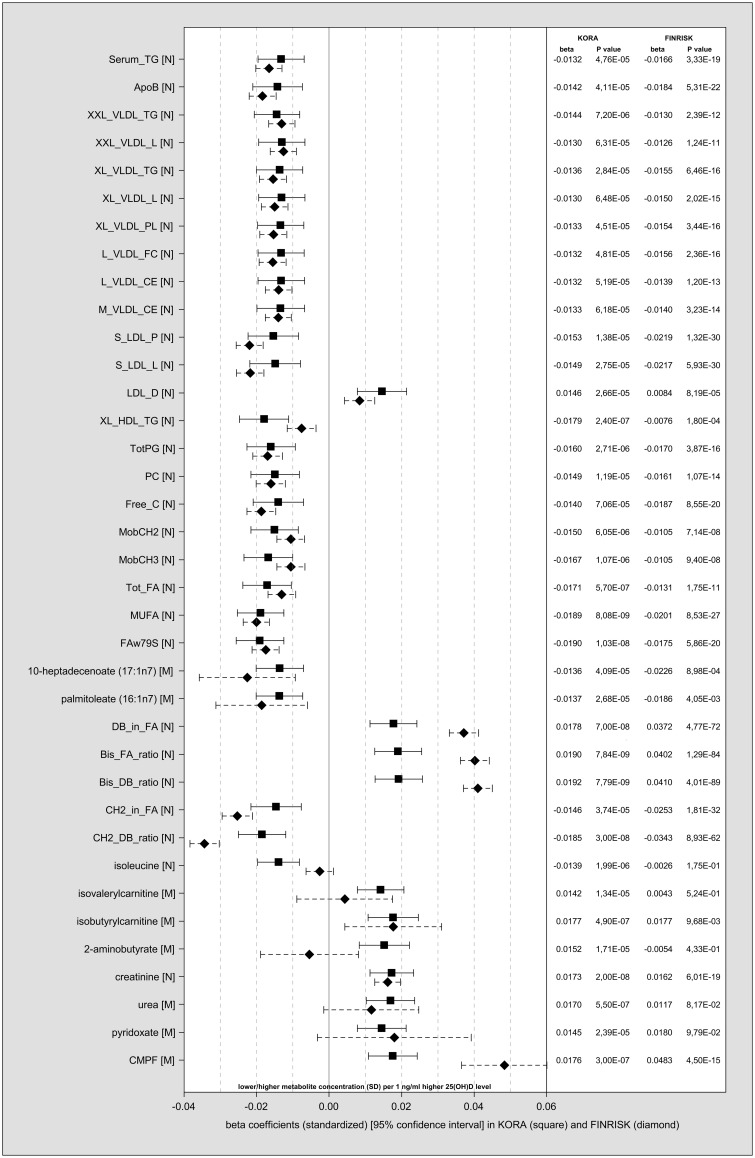
Individual metabolites associated with 25(OH)D in KORA F4 and FINRISK 1997. β-coefficients (standardized), 95% confidence intervals and *P*-values of the 37 metabolites associated with 25(OH)D (*P*-value threshold: 1.20 x 10^−4^) in KORA F4 (square). Of these, 30 associations were replicated in FINRISK 1997 (diamond) at *P* < 1.35 x 10^−3^. Main models: adjusted for age, sex, season (KORA F4 only), WC (continuous), physical activity, time outdoors (KORA F4 only), smoking status and alcohol consumption.

The results of the main analysis of the discovery step in KORA F4 for all metabolites are shown in Supplementary Table S4, available as Supplementary data at *IJE* online. Due to the standardization of the metabolites, the β-coefficients can be interpreted as the difference in metabolite concentration (in standard deviations) per 1 ng/ml higher 25(OH)D level. For serum triglycerides, for example, every 1 ng/ml higher 25(OH)D level was associated with a 0.0132 standard deviation lower mean triglyceride level. In total, 37 metabolites were associated with 25(OH)D at *P* < 1.20 x 10^−4^ in KORA F4, the majority inversely. For 30 of these metabolites, the association was replicated in FINRISK 1997 at *P* < 1.35 x 10^−3^ (Figure 1

). Theses metabolites cover: constituents of (very) large subclasses of very-low-density lipoprotein (VLDL) and small low-density lipoprotein (LDL) subclasses (all N) as well as related measures: serum triglycerides [Serum_TG (N)] and apolipoprotein B [ApoB ((N)]}; total fatty acids ([Tot_FA (N)]; the ω-7 monounsaturated long-chain fatty acid 10-heptadecenoate (17:1n7) (M); and various measures which reflect the degree of saturation of fatty acids [all (N) e.g. monounsaturated fatty acids (MUFA) ((N), ω-7, ω-9 and saturated fatty acids (FAw79S) ((N), the average number of double bonds in a fatty acid chain (DB)_in_FA (N)]; and the metabolic waste product creatinine (N); as well as with the urofuran acid 3-carboxy-4-methyl-5-propyl-2-furanpropanoate (CMPF) (M). For six of the non-replicated metabolites [the branched chain amino acid metabolites isoleucine (N), isovalerylcarnitine (M) and isobutyrylcarnitine (M), palmitoleate (16:1n7) (M), pyridoxate (M) (a metabolite of vitamin B6 metabolism) and urea (M)], the effect direction in FINRISK 1997 was the same as in KORA F4. Only 2-aminobutyrate (M) showed inconsistent effect directions, being positively associated with 25(OH)D in KORA F4, and negatively in FINRISK 1997.

### Individual metabolite associations in abdominally obese and non-obese participants

**Figure 2.**
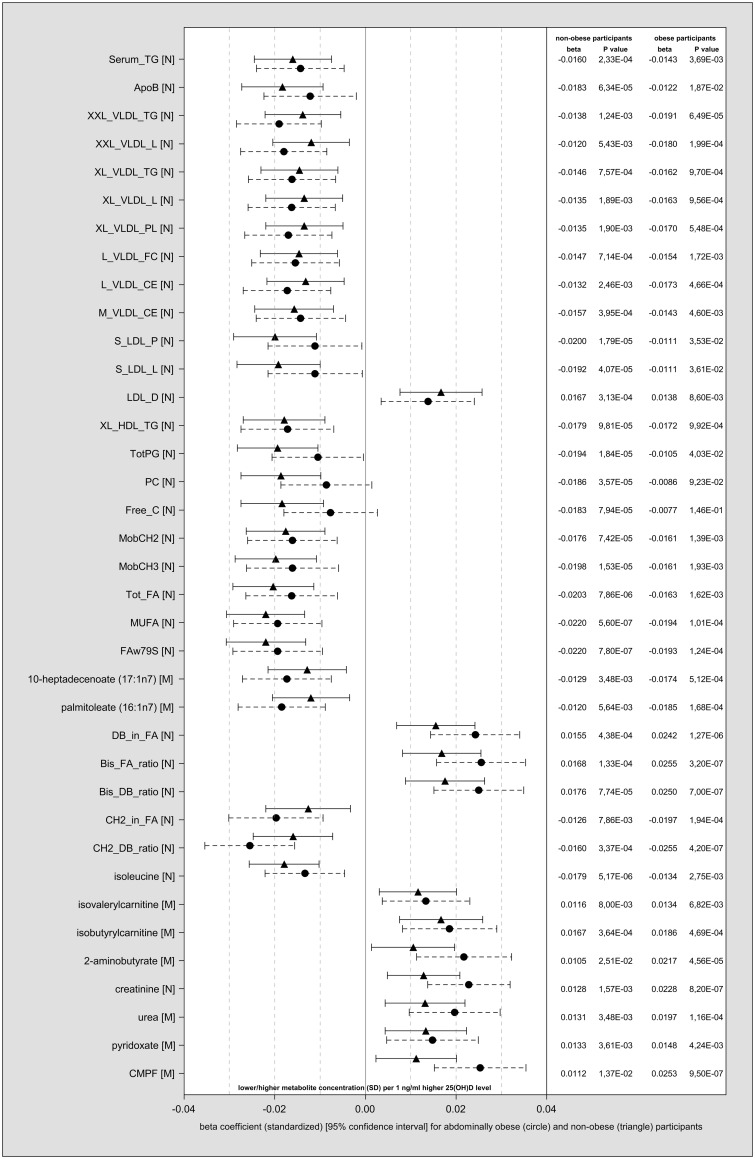
Individual metabolite associations in abdominally obese and non-obese participants in KORA F4. β-coefficients (standardized), 95% confidence intervals and *P*-values of the 37 metabolites associated with 25(OH)D from the main models for abdominally obese (circle) and non-obese (triangle) participants. In addition, 25(OH)D was associated with glucose (N) (β = −0.0204, *P*-value = 0.00004) in abdominally obese participants and with 3-methyl-2-oxovalerate (M) (β = −0.0162, *P*-value = 0.00005) in non-obese participants (*P*-value threshold was set at 6.02 x 10^−05^).

In general, the associations between 25(OH)D and the 37 metabolites observed at *P* < 1.20 x 10^−4^ in the main analyses were similar in abdominally obese and non-obese participants, both in KORA F4 (Figure 2

) and in FINRISK 1997. Exceptions in FINRISK 1997 were 2-aminobutyrate (M) and urea (M). For some metabolites, the strength of the association with 25(OH)D appeared to differ according to waist circumference status. For instance, the positive association of 25(OH)D with some measures of fatty acid polyunsaturation and with CMPF (M) seemed to be stronger in abdominally obese participants in both cohorts. However, none of the interaction terms in KORA F4 had a *P*-value < 6.02 x 10^−05^. In non-obese participants, 10 metabolites were inversely associated with 25(OH)D at *P* < 6.02 x 10^−05^ in KORA F4, eight of which were replicated in FINRISK 1997: small LDL particles, total lipids in small LDL (S_LDL_L) (N)) summary measures of fatty acids (Tot_FA) (N), MUFA (N), FAw79S (N), CH_3_ groups of mobile lipids (MobCH_3_) (N) and total phosphoglycerides, phosphatidylcholines. In abdominally obese participants, eight metabolites were associated with 25(OH)D at *P* < 6.02 x 10^−05^ in KORA F4, six of which were replicated in FINRISK 1997: the average number of methylene groups per double bond (CH_2__DB_ratio) (N) was inversely associated, DB_in_FA (N), the ratio of bisallylic groups to double bonds (Bis_DB_ratio) (N), the ratio of bisallylic groups to total fatty acids (Bis_FA_ratio) (N), creatinine (N) and CMPF (M) were positively associated.

### Sensitivity analyses in KORA F4

Additional characteristics of KORA F4 for the sensitivity analyses are shown in the Supplementary Table S5 (available as Supplementary data at *IJE* online) and a more detailed description of the results of the sensitivity analyses is given in the Supplementary material as well. Briefly, 25(OH)D levels were inversely associated with two of the metabolite modules: M1, which is mainly represented by total triglycerides, constituents of all VLDL subclasses and measures of primarily saturated and monounsaturated fatty acids (*P* = 0.00002); and M3, which is mainly represented by constituents of LDL and intermediate density lipoprotein (IDL) subclasses, measures of serum cholesterol and Apo B (*P* = 0.00332) (Supplementary Table S6, available as Supplementary data at *IJE* online). For the majority of the 37 metabolites that were associated with 25(OH)D at *P* < 1.20 x 10^−4^, a linear relationship seems likely (Supplementary Figure S3, available as Supplementary data at *IJE* online) and the tests for linear trends were significant at *P* < 1.35 x 10^−3^ for all 37 metabolites except Free_C (N) (data not shown). Using BMI instead of WC (Supplementary Figure S4, available as Supplementary data at *IJE* online) to operationalize obesity as well as additional adjustment for parathyroid hormone (Supplementary Table S7, available as Supplementary data at *IJE* online) resulted in only marginal changes in the effect estimates. Additional adjustment for health status (Supplementary Figure S6) and intake of medication (Supplementary Figure S7, available as Supplementary data at *IJE* online) resulted in additional constituents of VLDL subclasses being associated with 25(OH)D at *P* < 1.20 x 10^−4^, whereas the association with isobutyrylcarnitine (M), isovalerylcarnitine (M), pyridoxate (M), creatinine (N) and urea (M) disappeared.

## Discussion

In the present study, we used serum metabolomics data obtained from two analytical platforms to characterize the metabolic profile associated with serum 25(OH)D levels in 1726 participants of the population-based KORA F4 study. In total, 37 of the 415 examined metabolites were associated with 25(OH)D after Bonferroni correction. Of these, 30 associations were replicated in the population-based FINRISK 1997 study. These metabolites include constituents of (very) large VLDL and small LDL subclasses and related measures like serum triglycerides and ApoB, various summary measures of fatty acids and fatty acid polyunsaturation as well as CMPF. The observed associations were independent of waist circumference and were generally similar in abdominally obese and non-obese participants. Furthermore, they persisted after adjustment for parathyroid hormone, health status and intake of a variety of medications.

To our knowledge, this is the first study in which the serum metabolite profile associated with 25(OH)D levels is examined in such a comprehensive way. In a previous study, an NMR spectroscopy-based approach was used to identify a metabotype responsive to vitamin D supplementation with regard to markers of the metabolic syndrome.^20^ In the identified responsive metabotype, a 4-week vitamin D supplementation reduced VLDL and LDL, glucose and glycerol-phosphocholine in the nine participants of the intervention group.^20^ In another study, Finkelstein and colleagues examined the association between 25(OH)D levels and the serum metabolite profile (measured by Metabolon Inc.) in 30 pregnant adolescents.^21^ Using a hierarchical mixture model, they identified 11 metabolites which were associated with 25(OH)D levels ≥ 20 ng/ml: higher pyridoxate, bilirubin, xylose and cholate levels, as well as lower leukotrienes, 1,2-propanediol, azelate, undecanedioate, sebacate, inflammation-associated complement component 3 peptide (HWESASXX) and piperine.^21^ None of these metabolites was associated with 25(OH)D in the present study. This discrepancy may be due to differences in the study populations (middle-aged to older adults with pregnant participants being excluded, vs pregnant adolescents).

In the present study, higher 25(OH)D levels were associated with lower levels of serum triglycerides, constituents of (very) large VLDL subclasses and small LDL particles. Furthermore, levels of Apo B, the primary apolipoprotein in atherogenic lipoprotein particles (VLDL, IDL and LDL) and thus an estimate of total atherogenic particle number,^40^ were lower with higher 25(OH)D levels. High levels of triglycerides, VLDL and LDL, especially small LDL, are important risk factors for atherosclerosis and coronary heart disease.^41^ Atherogenic dyslipidaemia, a triad of increased concentrations of small, dense LDL particles, decreased high-density lipoprotein particles and increased triglycerides, is a characteristic of type 2 diabetes, insulin resistance and the metabolic syndrome and an important risk factor for myocardial infarction and cardiovascular disease.^42^ An underlying mechanism of the inverse association between 25(OH)D levels and atherogenic lipids observed in the present study might be the relationship between vitamin D and lipoprotein lipase (LPL), an enzyme that catalyses the lipolysis of triglycerides in lipoproteins into two free fatty acids and one monoacylglycerol^43^ and thereby converts VLDL to IDL and then to LDL.^41^ Serum levels of 25(OH)D were found to be positively associated with LPL concentrations in a cohort of Chinese adults^43^ and 1,25(OH)_2_D was found to increase lipoprotein lipase expression and activity *in vitro.*^44^ Higher 25(OH)D levels were associated with lower levels of total fatty acids as well as with an increased degree of polyunsaturation of fatty acids (e.g. with a higher ratio of bisallylic groups to total fatty acids^45^) in the present study. In part, this association is likely to reflect diet quality. In a recent clinical trial, a healthy diet characterized by increased intake of whole grain products, fish and bilberries, resulted in an increased degree of polyunsaturation of plasma fatty acids.^45^ Interestingly, however, this healthy diet in particular increased ω-3 fatty acids, whereas in our study, 25(OH)D was not associated with total ω-3 fatty acids (β = 0.0018, *P*-value = 0.62) or single ω-3 fatty acids. Thus, the positive association between 25(OH)D and fatty acid polyunsaturation observed in the present study may be more than a simple representation of diet quality. In participants of the Cardiovascular Risk in Young Finns Study, measures of increased fatty acid polyunsaturation were found to be associated with a decreased risk for 6-year high intima-media thickness progression and low 6-year carotid distensibility prevalence, both markers of subclinical atherosclerosis.^46^ Furthermore, the risk for low 6-year carotid distensibility prevalence was increased with higher levels of apolipoprotein B.^46^

Of note, the majority of the metabolites associated with 25(OH)D levels in the present study were also associated with weight change in our previous work.^32^ As a reduction in BMI is associated with increased levels of serum 25(OH)D,^47^ this speaks in favour of a confounding effect of obesity. However, the associations observed in the present study were independent of waist circumference or BMI and were generally similar in abdominally obese and non-obese participants. In fact, the association with a cluster of metabolites constituting atherogenic lipids (metabolite module M3) was only found in non-obese participants. Thus, the associations observed in the present study are unlikely to merely reflect pathomechanisms associated with obesity.

Our study is among the first to use a metabolomics approach to examine 25(OH)D levels, and the results of this study are based on large study populations from phenotypically well-characterized population-based cohorts. However, several limitations need to be mentioned, of which the greatest is the cross-sectional design. Although we did adjust our models for a variety of socioeconomic, lifestyle and health-related variables, cross-sectional findings are prone to residual confounding. Specifically, associations with 25(OH)D might in fact reflect associations which are mediated by other bioactive components induced by ultraviolet radiation, such as nitric oxide.^48^ In male mice for example, long-term ultraviolet radiation, which had no influence on serum 25(OH)D levels, suppressed measures of the metabolic syndrome such as cholesterol levels.^48^ The observed associations might further be the result of a healthy lifestyle involving increased sun exposure due to more frequent outdoor physical activity and a healthy diet rich in vitamin D, which was not sufficiently captured by the covariables in our models. In particular, the measures of physical activity were quite imprecise and diet quality was not assessed in the KORA F4 study. The latter aspect might also be reflected in our results by the positive association of 25(OH)D with CMPF, which has recently been identified as a highly specific biomarker for fatty fish intake.^49^ The association with CMPF was considerably stronger in abdominally obese participants. Given that obese individuals are less likely to be exposed to sunlight, due to less involvement in outdoor activities and due to different clothing habits,^11^ their major source of vitamin D might be diet rather than exposure to sunlight. Due to the cross-sectional design, we cannot draw conclusions on the causality or direction of the observed associations. Thus, the inverse associations between 25(OH)D and the metabolites reflective of a cardiometabolic risk profile might indicate an underlying disease, for which a low 25(OH)D status merely serves as a biomarker.^50^ The KORA F4 participants are relatively healthy and the associations between 25(OH)D and the metabolites persisted after adjustment for health status and intake of a variety of medications. However Mendelian randomization studies, which should be unaffected by confounding and reverse causation,^51^ provide ambiguous results on the relationship between vitamin D and cardiometabolic health. Whereas some results support a causal effect of higher vitamin D levels on a more favourable lipid profile,^52^ other studies question the role of 25(OH)D in the aetiology of obesity^53^ or type 2 diabetes,^54^ ischaemic heart disease^55^ and cardiovascular mortality^56^. Furthermore, levels of 25(OH)D were relatively low in both cohorts. In KORA F4, this may be partly attributable to the use of the DiaSorin LIAISON assay. This assay has a high variance, especially at high concentrations, and compared with the gold-standard liquid chromatography-tandem mass spectrometry, it was found to underestimate 25(OH)D levels (mean bias: -7.2 ng/ml).^57^ A linear trend was observed for the association between 25(OH)D and the metabolites, but conclusions for 25(OH)D levels outside the observed range should still be drawn with care.

In conclusion, using serum metabolomics obtained from two MS- and NMR-spectroscopy-based platforms, we observed that higher serum 25(OH)D levels were associated with a metabolic profile characterized by lower concentrations of atherogenic lipids and a higher degree of fatty acid polyunsaturation. These associations, which reflect the well-observed relationship between 25(OH)D deficiency and cardiometabolic diseases, were independent of waist circumference and, in general, similar in abdominally obese and non-obese individuals. Therefore, our results indicate that the relationship between vitamin D deficiency and cardiometabolic diseases is unlikely to merely reflect pathomechanisms related to obesity. However, additional longitudinal studies are needed to validate these results before further conclusions on the biological mechanisms linked to vitamin D can be drawn.

## Supplementary Data

Supplementary data are available at *IJE* online.

## Funding

This work was supported by the Kompetenznetz Adipositas (Competence Network Obesity), funded by the German Federal Ministry of Education and Research [FKZ 01GI1121B]. The KORA study was initiated and financed by the Helmholtz Zentrum München–German Research Center for Environmental Health, which is funded by the German Federal Ministry of Education and Research (BMBF) and by the State of Bavaria. Furthermore, KORA research was supported within the Munich Center of Health Sciences (MC-Health), Ludwig-Maximilians-Universität, as part of LMUinnovativ. The serum NMR metabolomics was supported by Sigrid Juselius Foundation and Strategic Research Funding from the University of Oulu, Finland, and UK Medical Research Council (MC_UU_12013/1). This work was further supported by the European Union Seventh Framework Programme (FP7/2007-2013) under grant ‘The BiomarCaRE study agreement No. HEALTH-F2-2011-278913 (BiomarCaRE)'. J.K. was supported through funds from the Academy of Finland (grant number 283045). K.S. is supported by ‘Biomedical Research Program’ funds at Weill Cornell Medical College in Qatar, a programme funded by the Qatar Foundation. M.P. was supported by the EU FP7 under grant agreements no. 313010 (BBMRI-LPC), no. 305280 (MIMOmics), and HZ2020 633589 (Ageing with Elegance), the Finnish Academy (grant no. 269517), the Yrjö Jahnsson Foundation and the Juho Vainio Foundation. V.S. was supported by the Finnish Foundation for Cardiovascular Research.

**Conflict of interest**. S.B. has received research funding from Abbott, Abbott Diagnostics, Bayer, Boehringer Ingelheim, SIEMENS and Thermo Fisher and received honoraria for lectures from Abbott, Abbott Diagnostics, AstraZeneca, Bayer, Boehringer Ingelheim, Medtronic, Roche, SIEMENS, SIEMENS DX and Thermo Fisher. S.B. has also received honoraria as a member of Advisory Boards and for consulting for Boehringer Ingelheim, Bayer, Novartis, Roche and Thermo Fisher. P.S., A.J.K. and M.A-K. are shareholders of Brainshake Ltd, a company offering NMR-based metabolite profiling. J.K., P.S. and A.J.K. report employment relation with Brainshake Ltd. The other authors declare that they have no conflict of interest.

## Supplementary Material

Supplementary Data
