# Supplementary material for: Characterization of the metabolic profile associated with serum 25-hydroxyvitamin D: a cross-sectional analysis in population-based data
Source: Int J Epidemiol. 2016 Sep 7;45(5):1469–81. doi: 10.1093/ije/dyw222 (PMC5100623; doi:10.1093/ije/dyw222)
Supplement: Supplementary Data [file supp_45_5_1469__index.html]

Characterization of the metabolic profile associated with serum 25-hydroxyvitamin D: a cross-sectional analysis in population-based data — Characterization of the metabolic profile associated with serum 25-hydroxyvitamin D: a cross-sectional analysis in population-based data — Supplementary Data 

# Characterization of the metabolic profile associated with serum 25-hydroxyvitamin D: a cross-sectional analysis in population-based data

## Supplementary Data

files

- Supplementary Data - doc file
- Supplementary Data - xls file
