# Supplementary material for: Characterization of the metabolic profile associated with serum 25-hydroxyvitamin D: a cross-sectional analysis in population-based data
Source: Int J Epidemiol. 2016 Sep 7;45(5):1469–81. doi: 10.1093/ije/dyw222 (PMC5100623; doi:10.1093/ije/dyw222)
Supplement: Supplementary Data [file supp_dyw222_Additional_file_1_Supplementary_Material_and_Supplementary_Figures.doc]

Additional file 1: Supplementary Material

to

**Characterization of the metabolic profile associated with serum vitamin D**

A cross-sectional analysis in population-based data

Susanne Vogt^†^, Simone Wahl^†^, Johannes Kettunen, Susanne Breitner, Gabi Kastenmüller, Christian Gieger, Karsten Suhre, Melanie Waldenberger, Jürgen Kratzsch, Markus Perola, Veikko Salomaa, Stefan Blankenberg, Tanja Zeller, Pasi Soininen, Antti J Kangas, Annette Peters, Harald Grallert, Mika Ala-Korpela, Barbara Thorand*

^†^ Equal contributors

* Correspondence: thorand@helmholtz-muenchen.de

**Additional methods**

Handling of missing data in the discovery cohort KORA F4

After removal of metabolites with more than 50% missing values, the metabolomics data set contained a total of 39806 (5.6%) missing values (see Supplementary Table 3 for the extent of missing values per variable). Of the MS variables, 17 (5.7%) were completely observed, while the remaining 279 (94.3%) had missing entries. The median number of missing observations among MS variables was 15 (8.7%), with a range from 0 to 855 (49.5%). Of the NMR variables, seven (5.9%) were completely observed. The median number of missing observations was 6 (3.5%), with a range from 0 to 562 (32.6%). The distribution of missingness proportions among the metabolites is visualized in Supplementary Figure S1 Phenotypic variable contained almost no missing values, with a maximum of six missing values observed for body weight, and no missing values for 25(OH)D.

Multiple imputation by chained equations (MICE) was chosen for missing data treatment.[^1^](#_ENREF_1) The general concept of multiple imputation is that (1) data are imputed multiple times (in MICE, by a repeated chain of regression models through the incomplete variables, whereby information is obtained from other, potentially correlated variables in the data-set). (2) The statistical methodology of choice (in our case, regression modelling) is applied to each of the imputed data-sets. Finally, (3) the obtained results (i.e., regression coefficient estimates and their variances) are summarized across the imputations. In the case of regression coefficients, this is straight-forward, since Rubin’s rules apply.[^2^](#_ENREF_2) Rubin’s methodology ensures that the uncertainty from the unobserved values is acknowledged in the computation of the variance, thus avoiding the underestimation of variance observed for single imputation and ensuring the validity of the results. MICE was conducted using the R package *mice*, version 2.21.[^1^](#_ENREF_1) As the procedure was conducted exactly as described in our earlier work,[^3^](#_ENREF_3) a description of missingness in the KORA F4 data – including correlations between variables and between variables and missingness of variables – as well as details on imputation methodology and diagnostics, can be found there. The only difference to this previous work was the selection of phenotypic variables included in the imputation process.

Missingness of the metabolites did not strongly relate to 25(OH)D levels. Seven metabolites were significantly correlated with 25(OH)D levels after correction for multiple testing: biliverdin [M] (Kendall cor=-0.086), gamma-glutamyltyrosine [M] (cor=-0.085), glycylvaline [M] (cor=-0.086), leucylleucine [M] (cor=-0.088), hexadecanedioate [M] (cor=0.077), XXL_VLVL_P [N] (cor=0.092) and hyodeoxycholate [M] (cor=0.106), however, these were not among the metabolites that we report to be associated with 25(OH)D levels. According to van Buuren et al.[^1^](#_ENREF_1), we included all phenotypic variables in the imputation models that were part of the regression model, including relevant interactions with vitamin D, as well as auxiliary variables (i.e., metabolites that are correlated with concentration or missingness of the metabolite in question; for exact definition see [^3^](#_ENREF_3)). This strategy is recommended in order to achieve unbiased results and reduce the uncertainty about the missing values.[^1^](#_ENREF_1) The data set was imputed 20 times, providing sufficient precision of the results.[^3^](#_ENREF_3)

Prior to imputation, the distribution of the metabolites and continuous phenotypic variables was checked. We tested raw, natural log transformed, cubic root and square root transformed variables for normality using Shapiro-Wilk tests and chose the transformation that showed the smallest deviation from normality (see Supplementary Table 2).

Metabolite module analyses in the discovery cohort KORA F4

A number of the metabolites, such as the lipoprotein subclasses, were highly correlated. Thus, in addition to the individual metabolite analyses, we employed a clustering approach to study the metabolic signatures associated with vitamin D levels. The cluster analysis was carried out analogously to our previous work.[^3^](#_ENREF_3) Thus, a detailed description of the clustering approach can be found there. Briefly, 411 serum metabolites and derived measures (281 from the MS and 130 from the NMR spectroscopy platform, respectively) were clustered into eight groups, so-called modules, of highly correlated molecules, using weighted correlation network analysis (WGCNA; R package *WGCNA*, version 1.34). For each of these eight metabolite modules (M1-M8), the module eigengene (ME), that is, the first principal component of a principal component analysis on the scaled matrix of molecules assigned to the respective modules, was determined as the ‘centre‘ of the module. The principal component weights, that is, the contributions of each metabolite to the respective ME (membership strength), were reused from.[^3^](#_ENREF_3) Since data were multiply imputed, MEs had to be computed for each of the 20 imputed data sets. In order to assess the relation of each module to 25(OH)D, we determined the association of the respective ME (outcome) with 25(OH)D levels (predictor) in linear models adjusted as described for the individual metabolites above in each of the imputed data sets, followed by combination across the imputed data sets.[^2^](#_ENREF_2) The adjusted *P* value threshold was set at 0.006 (0.05/8). In accordance with the individual metabolite analyses, we also explored associations stratified by WC status (adjusted *P* value threshold: 0.05/(2*8) = 0.003).

The module membership and module membership strength for each metabolite is shown in Supplementary Table S4.

**Additional results**

Metabolite module analyses

25(OH)D levels were inversely associated with two of the eight modules, M1 (*P* = 0.00002) and M3 (*P* = 0.00332) (Supplementary Table S6). M1 comprised 60 metabolites and is mainly represented by total triglycerides, constituents of all VLDL subclasses and measures of primarily saturated and monounsaturated fatty acids. M3 comprised 39 metabolites and is mainly represented by constituents of LDL and intermediate density lipoprotein subclasses, measures of serum cholesterol and Apo B. While both M1 and M3 were associated with 25(OH)D in non-obese participants, only M1 was associated with 25(OH)D in abdominally obese participants (Supplementary Table S6).

To assess whether M1 and M3 are independent in their association with 25(OH)D, we repeated the analyses using log transformed 25(OH)D levels as the outcome variables and the metabolite modules M1 and M3 as the predictor variables (adjusted for age, sex, season, waist circumference, physical activity, time spend outdoors, smoking status and alcohol). Only the association with M1 remained (*P* = 0.00063), while M3 was no longer associated with 25(OH)D (*P* = 0.685).

Sensitivity analyses

Additional adjustment of the main models for parathyroid hormone (Supplementary Table S7) resulted in only marginal changes in the effect estimates. With the exception of isovalerylcarnitine [M], all of the 37 metabolites which were associated with 25(OH)D in the main analyses, were also associated with 25(OH)D (at *P* < 1.20 x 10^-4^) in the PTH models. Moreover, 13 metabolites were additionally associated with 25(OH)D at *P* < 1.20 x 10^-4^.

Using BMI instead of WC to operationalize obesity resulted in 43 metabolites being associated with 25(OH)D (*P* < 1.20 x 10^-4^, results shown in Supplementary Figure 4). These included the 37 metabolites which were associated with 25(OH)D in the main analysis as well as six additional metabolites, which were already borderline significant in the main analysis (*P* values ranging from 1.29 x 10^-04^ to 5.65 x 10^-04^) and either clustered in M1 or M3. Furthermore, the effect estimates of all metabolites were quite similar in the WC and the BMI models (Supplementary Figure 5). Thus, the obesity operationalization did not severely affect the results. Additional adjustment of the main models for health status (disease model, results shown in Supplementary Figure 6) resulted in 42 metabolites being associated with 25(OH)D at *P* < 1.20 x 10^-4^. In comparison with the main models, additional constituents of VLDL subclasses were associated with 25(OH)D, while the association with isobutyrylcarnitine [M], isovalerylcarnitine [M], pyridoxate [M] and with the metabolic waste products creatinine [N] and urea [M] disappeared. Additional adjustment of the disease models for intake of medication (medication model, results shown in Supplementary Figure 7) resulted in 42 metabolites being associated with 25(OH)D at *P* < 1.20 x 10^-4^. In comparison with disease models, cholesterol [M] and S_LDL_C [N] were not associated with 25(OH)D anymore, while 3-methyl-2-oxovalerate [M] and the number of double bond protons of mobile lipids was additionally associated. A seasonal variation in the metabolite concentrations (at *P* < 1.20 x 10^-4^) independent of 25(OH)D levels was found for 27 metabolites (Supplementary Table S8), 14 of which were among the 37 metabolites associated with 25(OH)D at *P* < 1.20 x 10^-4^ (Bis_DB_ratio [N], CH2_DB_ratio [N] DB_in_FA [N], FAw79S [N], isoleucine [N], L_VLDL_CE [N], L_VLDL_FC [N], LDL_D [N], urea [M], XL_VLDL_L [N], XL_VLDL_PL [N], XL_VLDL_TG [N], XXL_VLDL_L [N], XXL_VLDL_TG [N]). The other 13 metabolites were acetoacetate [N], alanine [N], albumin [N], glycine [N], lactate [N], leucine [N], M_HDL_CE [N], M_HDL_FC [N], M_HDL_L [N], pyruvate [N], S_HDL_L [N], S_HDL_P [N] and XXL_VLDL_PL [N].

**Supplementary Figure 1** Distribution of missingness proportions among the metabolites

**Supplementary Figure 2a** Distribution of serum 25(OH)D levels in KORA F4


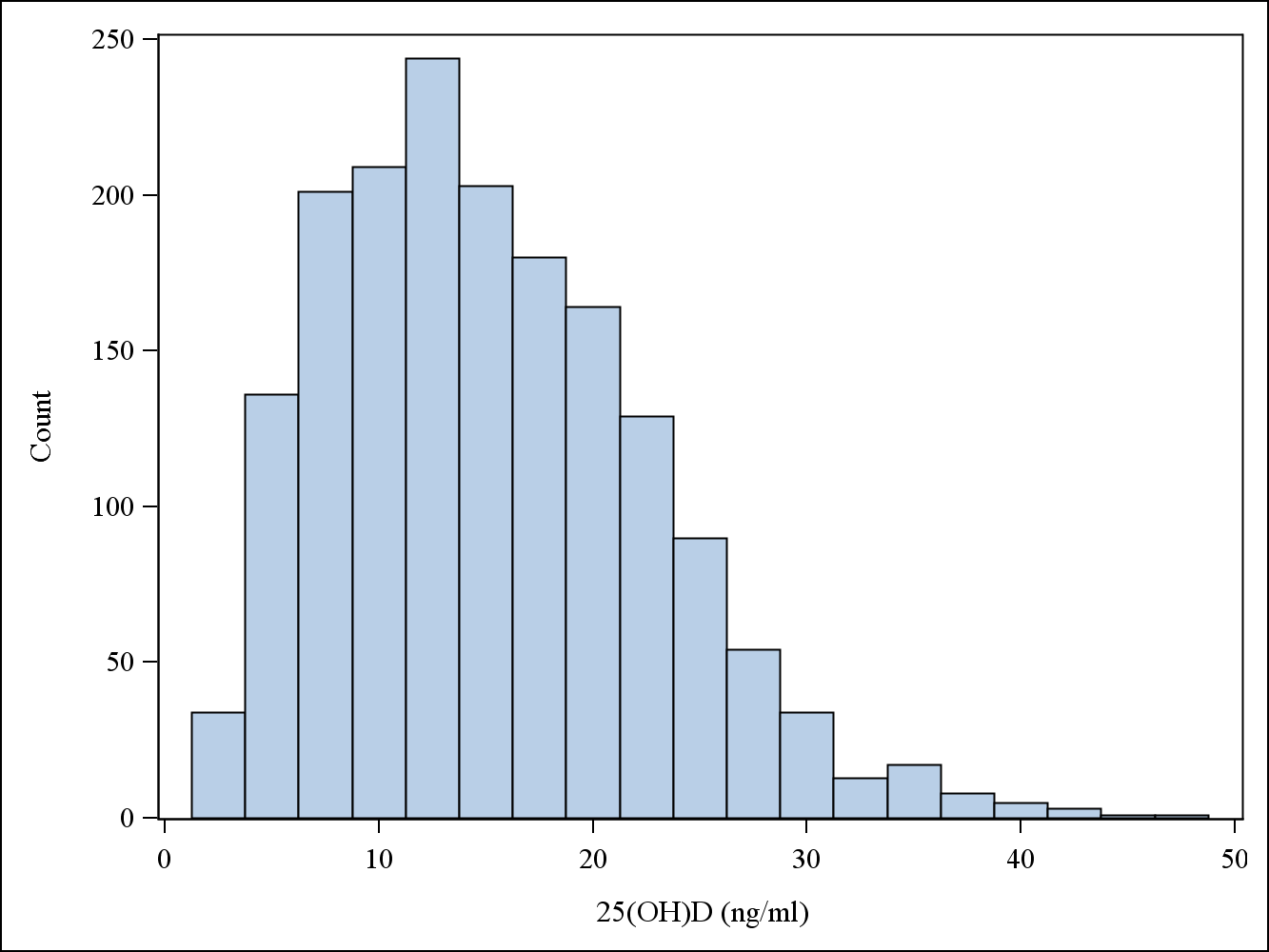


**Supplementary Figure 2b** Distribution of serum 25(OH)D levels in FINRISK 1997

**Supplementary Figure 3** Individual metabolite associations – 25(OH)D in quartiles


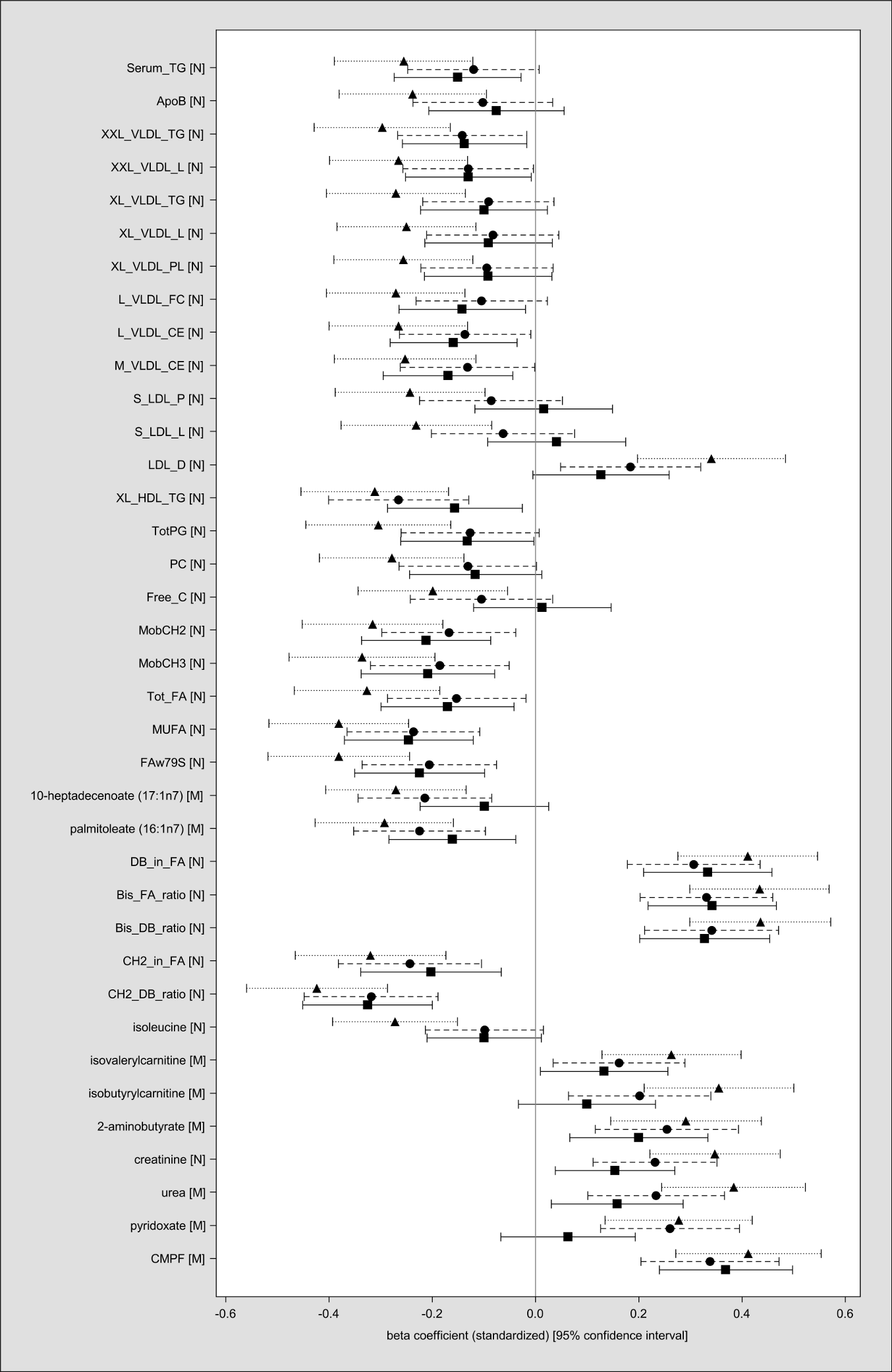


Standardized β-coefficients, 95% confidence intervals and *P* values of the 37 metabolites associated with 25(OH)D at *P* < 1.20 x 10^-4^ in the main analysis, using 25(OH)D levels in quartiles. The square represents quartile 2 (9.5-14.2 ng/ml), the circle represents quartile 3 (14.2-20 ng/ml ), the triangle represents quartile 4 (20-46.9 ng/ml), with quartile 1 (2.0-9.5 ng/ml) being the reference category.

**Supplementary Figure 4** Individual metabolite associations – BMI model


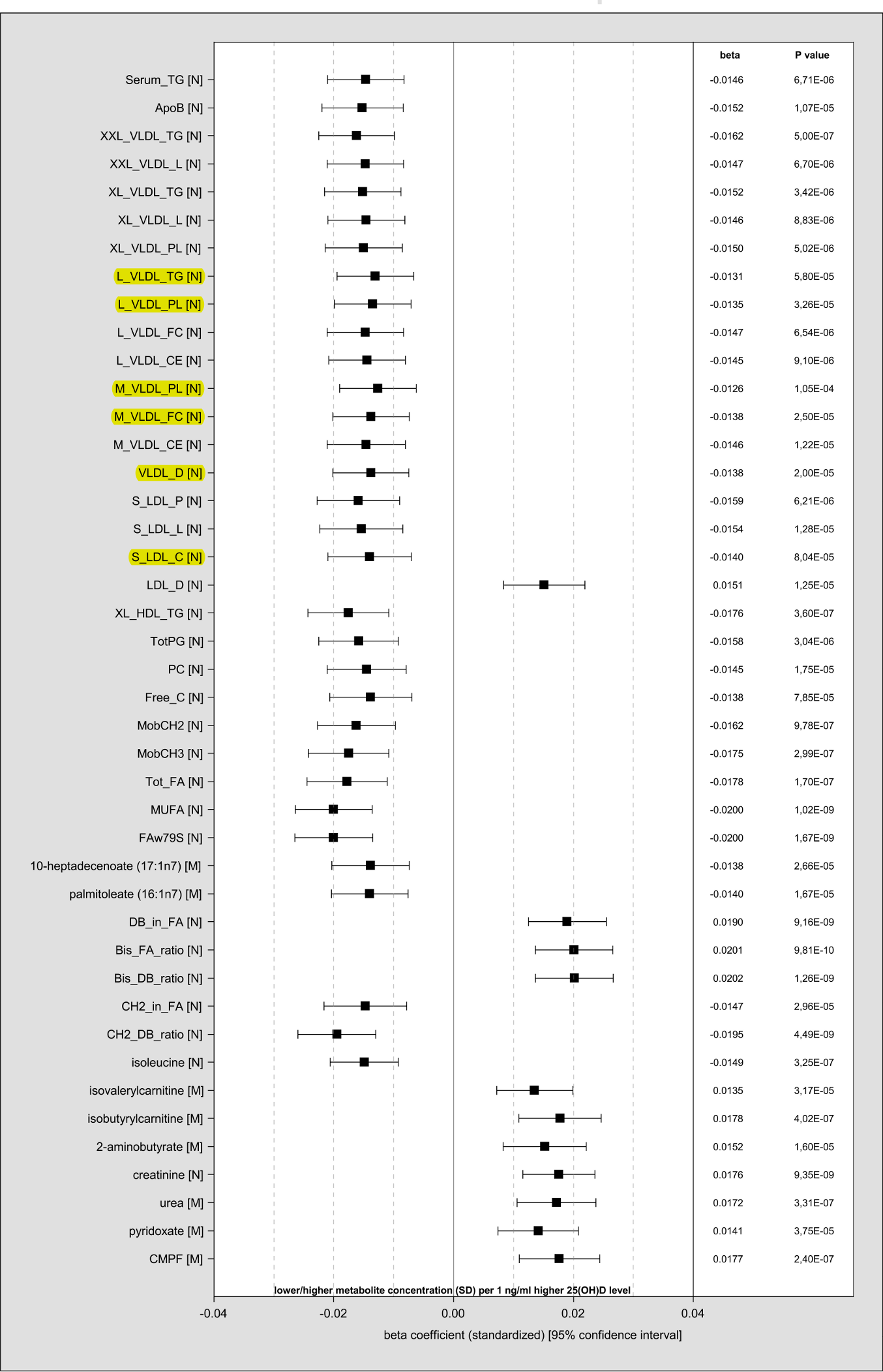


Standardized β-coefficients, 95% confidence intervals and *P* values of the 43 metabolites associated with 25(OH)D (*P* value threshold:1.20 x 10^-4^).

Models adjusted for age, sex, season, BMI (continuous), physical activity, time outdoors, smoking status and alcohol consumption

Metabolites, which were not associated with 25(OH)D in the main models, are marked in yellow.

**Supplementary Figure 5** Comparison of effect estimates of the WC and the BMI models


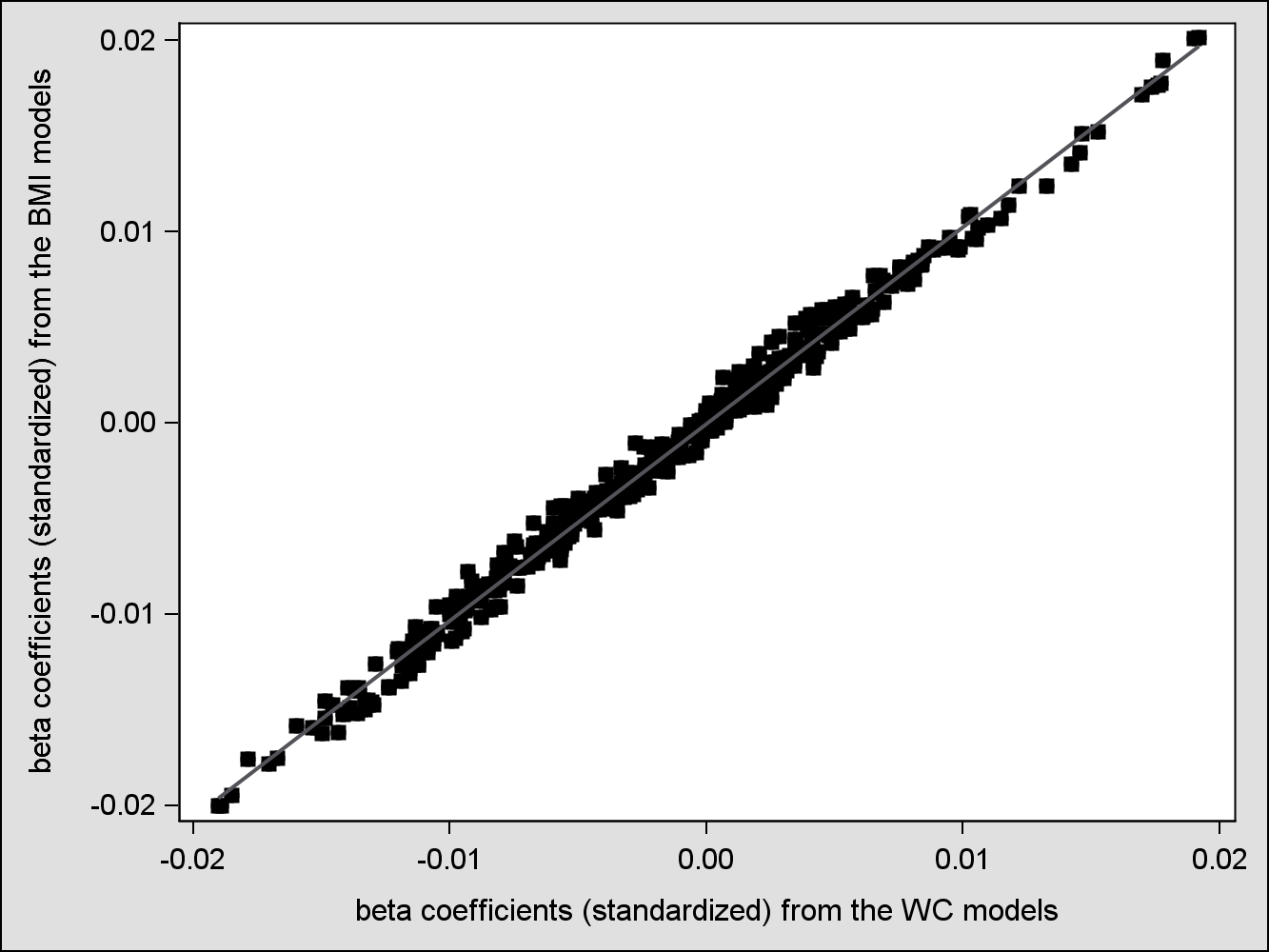


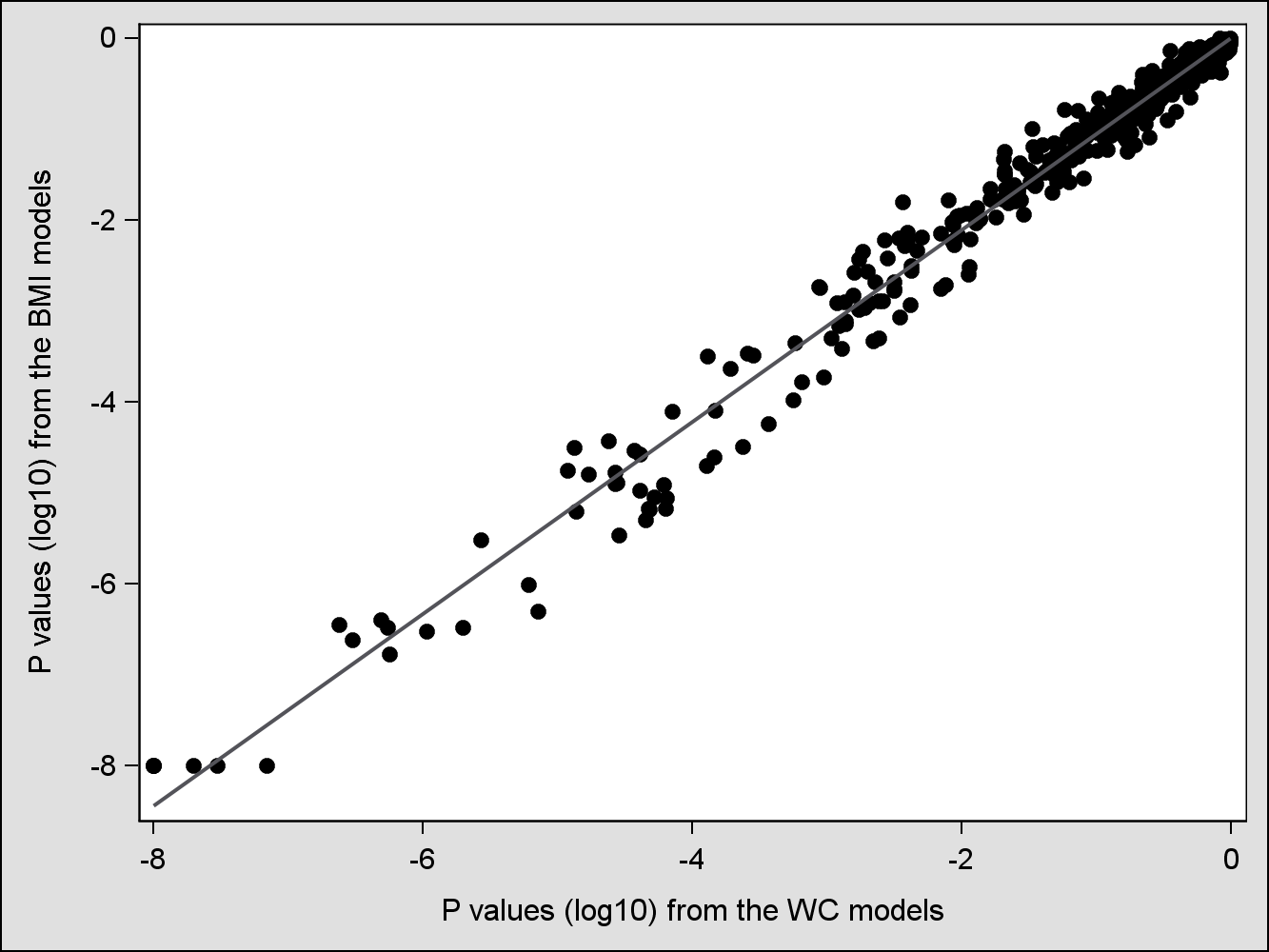


**Supplementary Figure 6** Individual metabolite associations – Disease model


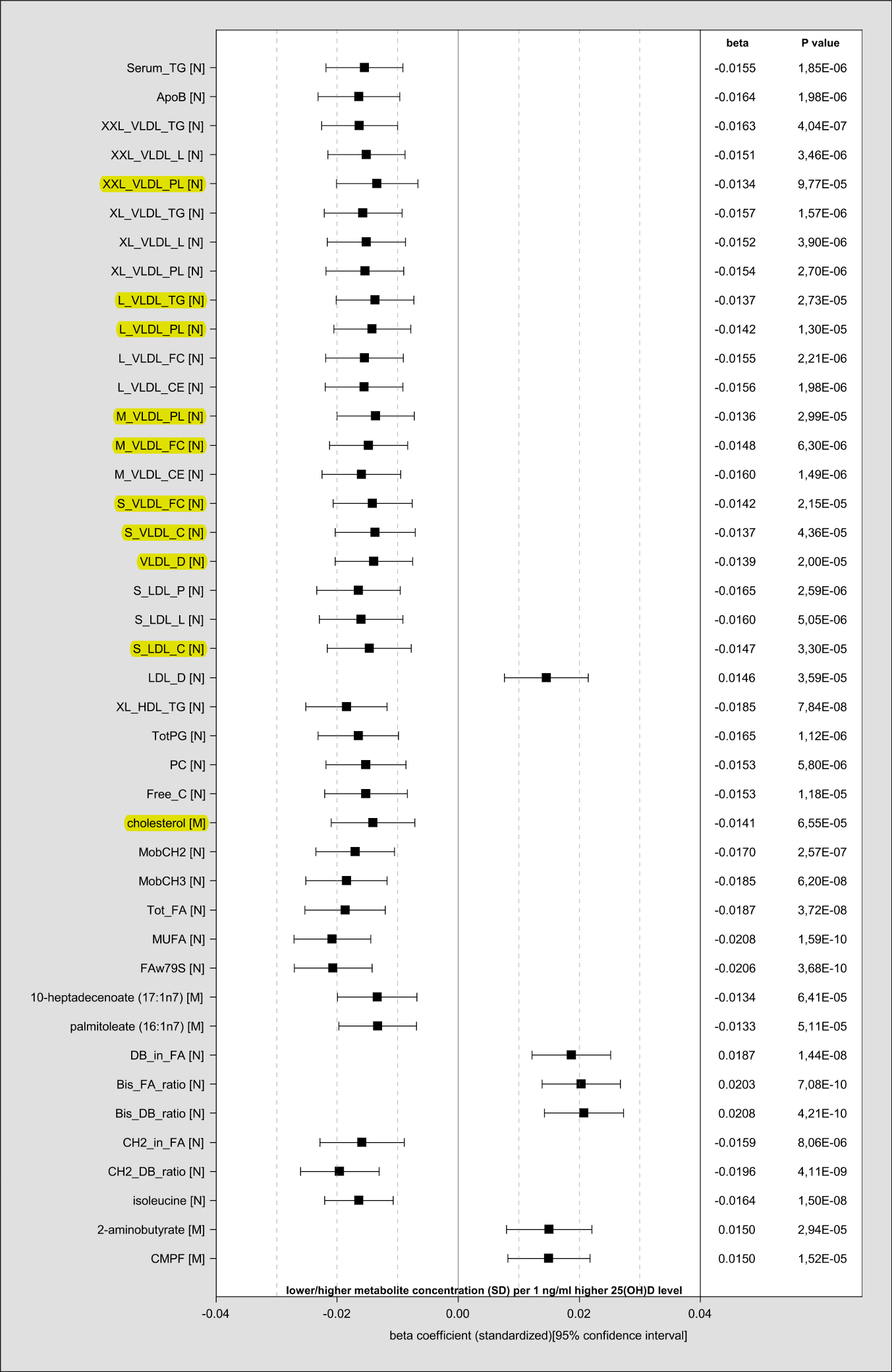


Standardized β-coefficients, 95% confidence intervals and *P* values of the 42 metabolites associated with 25(OH)D (*P* value threshold: 1.20 x 10^-4^).

Models adjusted for age, sex, season, WC (continuous), physical activity, time outdoors, smoking status and alcohol consumption, diabetes mellitus, history of myocardial infarction, stroke or cancer, self-rated physical constitution, estimated glomerular filtration rate, gamma-glutamyl transferase.

Metabolites, which were not associated with 25(OH)D in the main models, are marked in yellow. Isobutyrylcarnitine [M], isovalerylcarnitine [M], pyridoxate [M], creatinine [N] and urea [M] were not associated with 25(OH)D anymore.

**Supplementary Figure 7** Individual associations – Medication model


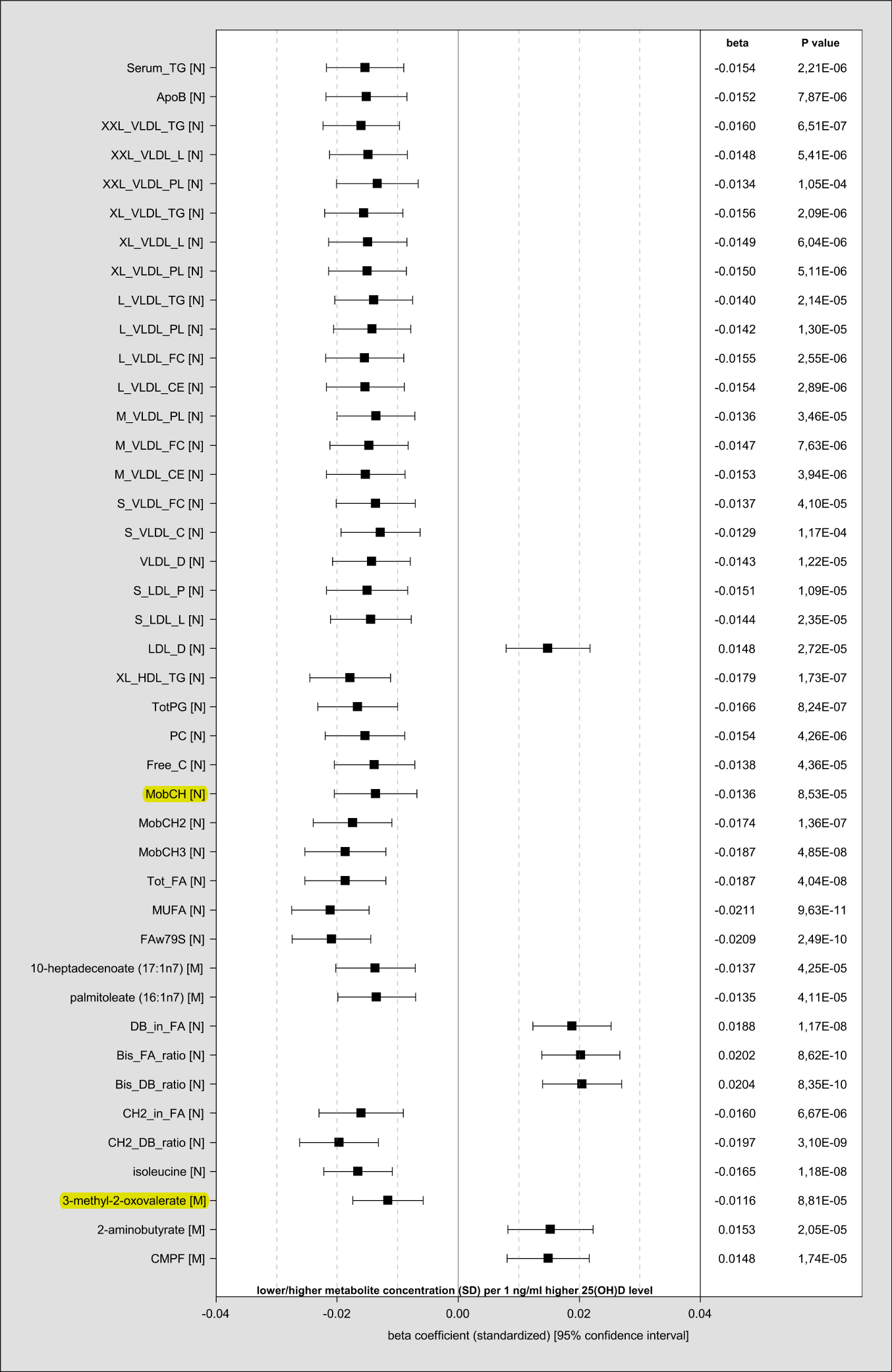


Standardized β-coefficients, 95% confidence intervals and *P* values of the 42 metabolites associated with 25(OH)D (*P* value threshold: 1.20 x 10^-4^).

Models adjusted for age, sex, season, WC (continuous), physical activity, time outdoors, smoking status and alcohol consumption, diabetes mellitus, history of myocardial infarction, stroke or cancer, self-rated physical constitution, intake of oral contraceptives, estrogens, diuretics, statins, antiepileptic and oral antidiabetic medication, corticoids, calcium channel blockers as well as angiotensin converting enzyme inhibitors or angiotensin antagonists.

Metabolites, which were not associated with 25(OH)D in the disease models, are marked in yellow. Cholesterol [M] and S_LDL_C [N] were not associated with 25(OH)D anymore.

**Additional References**

1. van Buuren S, Groothuis-Oudshoorn K. mice: Multivariate Imputation by Chained Equations in R. *Journal of Statistical Software* 2011; **45**.

2. Rubin DB. *Multiple Imputation for Nonresponse in Surveys*. New York, NY, USA: John Wiley & Sons, 1987.

3. Wahl S, Vogt S, Stuckler F, et al. Multi-omic signature of body weight change: results from a population-based cohort study. *BMC medicine* 2015; **13**: 48.

4. Zhang B, Horvath S. A General Framework for Weighted Gene Co-expression Network Analysis. In: Departments of Human Genetics and Biostatistics U, editor. *Statistical Applications in Genetics and Molecular Biology*. Los Angeles, CA, USA, 2005.

5. Langfelder P, Horvath S. WGCNA: an R package for weighted correlation network analysis. *BMC bioinformatics* 2008; **9**: 559.
